# BUILDING OUR LARGEST DEMENTIA INFRASTRUCTURE FOR ALZHEIMER'S ACT: POLICIES ADVANCING HEALTHY AGING

**DOI:** 10.1093/geroni/igac059.719

**Published:** 2022-12-20

**Authors:** Lisa McGuire

**Affiliations:** CDC, Atlanta, Georgia, United States

## Abstract

While public health experts cannot yet say how to prevent Alzheimer’s disease and related dementias (ADRD), emerging science indicates that ADRD may be slowed through risk reduction strategies, early diagnosis, and better education and training of front-line health care professionals. Recognizing these scientific advances, the National Plan to Address Alzheimer’s Disease: 2021 Update added a new goal, Goal 6, to the 10-year-old plan emphasizing risk reduction and healthy aging: Accelerate Action to Promote Healthy Aging and Reduce Risk Factors for Alzheimer’s Disease and Related Dementias. CDC is authorized through Building Our Largest Dementia (BOLD) Infrastructure for Alzheimer's Act (P.L. 115-406) to create a uniform national public health infrastructure through BOLD Program and Public Health Centers of Excellence. This presentation will highlight CDC activities that not only build a uniform national public health infrastructure but also promote healthy aging consistent with Goal 6 of the 2021 National Plan.

